# Magnetic Damping and Dzyaloshinskii–Moriya Interactions in Pt/Co_2_FeAl/MgO Systems Grown on Si and MgO Substrates

**DOI:** 10.3390/ma16041388

**Published:** 2023-02-07

**Authors:** Nabil Challab, Yves Roussigné, Salim Mourad Chérif, Mihai Gabor, Mohamed Belmeguenai

**Affiliations:** 1 LSPM–CNRS, UPR 3407, Université Sorbonne Paris Nord, F-93430 Villetaneuse, France; 2 Center for Superconductivity, Spintronics and Surface Science, Physics and Chemistry Department, Technical University of Cluj-Napoca, Str. Memorandumului No. 28, RO-400114 Cluj-Napoca, Romania

**Keywords:** Gilbert damping, magnetization relaxation, spin pumping, Heusler alloys, interfacial Dzyaloshinskii–Moriya interaction, perpendicular magnetic anisotropy

## Abstract

Spin-pumping-induced damping and interfacial Dzyaloshinskii–Moriya interaction (iDMI) have been studied in Pt/Co_2_FeAl/MgO systems grown on Si or MgO substrates as a function of Pt and Co_2_FeAl (CFA) thicknesses. For this, we combined vibrating sample magnetometry (VSM), microstrip ferromagnetic resonance (MS-FMR), and Brillouin light scattering (BLS). VSM measurements of the magnetic moment at saturation per unit area revealed the absence of a magnetic dead layer in both systems, with a higher magnetization at saturation obtained for CFA grown on MgO. The key parameters governing the spin-dependent transport through the Pt/CFA interface, including the spin mixing conductance and the spin diffusion length, have been determined from the CFA and the Pt thickness dependence of the damping. BLS has been used to measure the spin wave non-reciprocity via the frequency mismatch between the Stokes and anti-Stokes lines. iDMI has been separated from the contribution of the interface perpendicular anisotropy difference between Pt/CFA and CFA/MgO. Our investigation revealed that both iDMI strength and spin pumping efficiency are higher for CFA-based systems grown on MgO due to its epitaxial growth confirmed by MS-FMR measurements of the in-plane magnetic anisotropy. This suggests that CFA grown on MgO could be a promising material candidate as a spin injection source via spin pumping and for other spintronic applications.

## 1. Introduction

Spintronic devices are expected to be faster, denser, more efficient, and to operate with reduced power consumption [1]. For this, the magnetic material parameters must be well defined and tuned. Magnetic damping, characterized by the parameter α and usually referred to as the damping constant, spin polarization and magnetic anisotropy are key parameters for these spintronic devices functionality. Indeed, damping determines the switching current threshold of spin transfer torque-based devices [2], domain wall motion current densities [3], skyrmion velocities and strongly affects the direction of its motion [4]. It also affects the efficiency of magnonic devices since it is correlated to the spin wave diffusion length [5]. Therefore, the development of new materials with high spin polarization, optimized intrinsic damping, and tunable magnetic anisotropy is highly needed. Heusler alloys [6] are a promising group of materials to achieve low damping in metallic ferromagnets [7,8] used in charge-based spintronic devices. Indeed, these alloys are theoretically predicted to be half-metals (perfectly spin-polarized conduction electrons at Fermi energy) at room temperature, resulting in exceptional performances in spintronic devices. Since the intrinsic Gilbert damping has been shown to be proportional to the density of states at the Fermi level [9,10], Heusler alloys are expected to have extremely low damping compared to other conductive materials. Moreover, they have the advantage of a good lattice matching with major substrates, high Curie temperature and intermetallic controllability for spin density of states at the Fermi energy through the substitution of constituent elements of the alloy [11]. Due to its high Curie temperature (1000 K) and high spin polarization, Co_2_FeAl (CFA) is considered as an interesting candidate for spintronic applications.

Recently, it has been found that the ferromagnetic metal (FM)/heavy metal (HM) interface hosts a wide range of useful spin–orbit coupling-related phenomena, such as the spin Hall effect (SHE) [12] and the interfacial Dzyaloshinskii–Moriya interaction (iDMI) [13], which play an essential role in the development of a new generation of spintronic devices with boosted performances. In such a FM/HM system, the large spin–orbit interaction of the HM layer significantly increases the damping by spin-pumping [14]. Indeed, the precession of the magnetization induces a spin current that propagates from the FM across the FM/HM interface and dissipates in the HM layer, giving rise to an increased damping in the system and a transverse charge current via inverse SHE [15]. The key factor determining the spin transmission and the spin backflow through the FM/HM interface is the effective spin-mixing conductance $g_{eff}^{↑↓}$. The latter can be determined by measuring the FM thickness dependence of the damping constant of the in-plane magnetized system. Inversely, spin dynamics can be manipulated via a spin–orbit torque [16] by a spin injection into the FM generated by the flow of an electrical current in the plane of the HM using the SHE. Furthermore, iDMI is an antisymmetric exchange interaction which gives rise to non-collinear spin structures stabilizing chiral magnetization configurations with a nontrivial topology, which are important in the development of future spintronic devices. Therefore, the aim of this paper concerns the investigation of the spin-pumping-induced damping and the iDMI in Pt/Co_2_FeAl/MgO-based systems. The idea is to control their strength via optimizing the interface conditions through the variation of Co_2_FeAl and Pt thicknesses, as well as Co_2_FeAl crystal orientation. For this purpose, ferromagnetic resonance (FMR), Brillouin light scattering (BLS), and vibrating sample magnetometry (VSM) techniques were used. Our investigation shows that both iDMI and effective spin mixing conductance of epitaxial Co_2_FeAl films are higher compared to those of polycrystalline ones.

## 2. Samples and Methods

The samples used in this investigation were grown in a magnetron sputtering system with a base pressure lower than $3\times 10^{-8}$ Torr on Si (polycrystalline samples) and MgO (epitaxial films) substrates. The metallic films are DC sputtered in 1 mTorr of Ar working gas pressure, while the MgO films are rf sputtered at 10 mTorr. The epitaxial samples are grown on (001) MgO single crystal substrates and have the structure MgO//MgO (4)/Pd (2)/Pt (*t_Pt_*)/CFA (*t_CFA_*)/MgO (1)/Ta (2.5), where the number in parentheses represents the thickness in nm. The 2 nm Pd layer, grown directly on MgO substrate using a 4 nm thick MgO capping layer, is used to promote the epitaxial growth of the subsequent layers [17]. The Ta (2.5) capping layer protects the active structure from oxidation during exposure to the atmosphere. The polycrystalline samples are grown on thermally oxidized Si/SiO_2_ substrates and have the structure Si/SiO_2_//Ta (2.5)/Pd (2)/Pt (*t_Pt_*)/CFA (*t_CFA_*)/MgO (1)/Ta (2.5). The 2.5 nm Ta layer directly grown on Si/SiO_2_ is amorphous and facilitates the polycrystalline growth of the upper layers. For both systems, Pt and CFA thicknesses were varied (0.5 nm ≤ *t_Pt_* ≤ 8 nm and 1 nm ≤ *t_CFA_* ≤ 10 nm). When *t_Pt_* (*t_CFA_*) is varied, *t_CFA_* (*t_Pt_*) is fixed at 4 nm.

For each sample, the hysteresis loops were measured under the in-plane and the perpendicularl to the film plane applied magnetic fields using a VSM. The magnetic moment at saturation is then determined and used to obtain the saturation magnetic moment per unit area (*M_S_* × *t_CFA_*) for samples with variable *t_CFA_* and nominal magnetization at saturation for those with variable *t_Pt_*. Sweep-field microstrip ferromagnetic resonance (MS-FMR) [18], where the external applied magnetic field (up to 1.6 T) is modulated at 170 Hz by a small (4 Oe) alternating magnetic field, was used to determine the gyromagnetic ratio and the damping. For this, the frequency variation versus the perpendicular to the film plane applied magnetic field and the frequency dependence of the in-plane FMR field linewidth have been measured. For all performed MS-FMR measurements, the recorded spectra were fitted by a Lorentzian derivative function for each in-plane and perpendicular applied magnetic field to obtain the FMR resonance fields (*H_r_*), and the half linewidth at half maximum (∆*H*). To investigate the iDMI, BLS [19], under an in-plane applied magnetic field in Damon–Eshbach configuration, was employed to record spectra for a given field and spin wave vector. The Lorentzian fits of the spectra were used to obtain the Stokes (S) and anti-Stokes (aS) line frequencies, the frequency mismatch (∆*F* = *F_S_* − *F_aS_*) and, hence, the iDMI strength. 

## 3. Results and Discussion

### 3.1. Structural and Static Magnetic Properties

**Figure 1:**
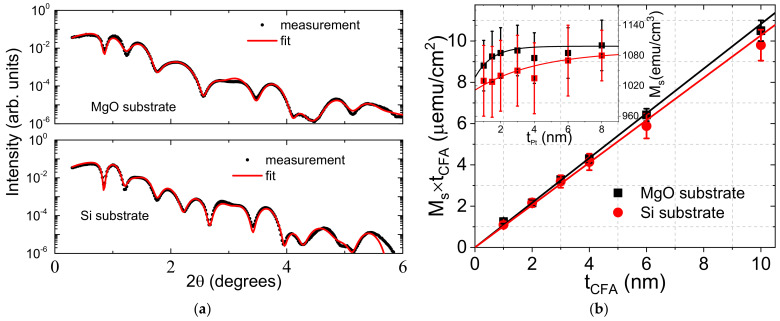
(**a**) X-ray reflectivity patterns of Pt (4 nm)/Co_2_FeAl (10 nm)/MgO systems grown on MgO or Si substrate. Experimental data are shown with solid circles while solid red lines are fits. (**b**) Variation in the saturation magnetic moment per unit area versus the CFA thickness (*t_CFA_*) for Pt (4 nm)/Co_2_FeAl (*t_CFA_*)/MgO systems grown on MgO or Si substrate. Symbols are VSM measurements and lines refer to the linear fits. The inset shows the corresponding Pt thickness dependence of the magnetization at saturation of Pt (*t_Pt_*)/Co_2_FeAl (4 nm)/MgO, where symbols refer to experimental data and solid lines are fits with the phenomenological equation indicated in the text.

In the case of the samples grown on MgO, CFA films grew according to the CFA(001)[110]//MgO(001)[100] epitaxial relation while those deposited on Si show a (011) fiber-texture with no in-plane preferential growth direction. Furthermore, CFA films with thicknesses below 10 nm are in the A2 phase, characterized by a complete chemical disorder between the Fe, Co, and Al sites. [17,20,21,22]. Moreover, the quality of the multilayer was assessed by X-ray reflectometry (XRR) measurements. The typical XRR patterns are shown in Figure 1a for Pt (4 nm)/Co_2_FeAl (10 nm)/MgO grown on Si/SiO_2_ or MgO substrate along with fits to the experimental data within the Parratt formalism. For all the layers, the maximum thickness deviations from the nominal values were lower than 7% and the roughness was below 0.7 nm. The relatively long attenuation length and reduced oscillations decay implies an overall low roughness and good interface sharpness, attesting to the high quality of the stacks.

The CFA thickness dependence of the saturation magnetic moment per unit area (M_S_ × *t_CFA_*) is shown in Figure 1b for both systems. It allows for the deduction of a zero-magnetic dead layer and a magnetization at saturation (*M_S_*) of 1083 ± 20 emu/cm^3^ and 1027 ± 20 emu/cm^3^ for films grown on MgO and Si substrates, respectively. The slightly higher *M_S_* of CFA grown on MgO can be attributed to the improved crystallinity of this system. Furthermore, while the absence of a magnetic dead layer agrees with the previously reported values [23], the *M_S_* values are significantly higher, probably due to proximity-induced magnetization (PIM) at the Pt/Co_2_FeAl interface. This seems to be sustained by the increase in the nominal *M_S_* with Pt thickness (*t_Pt_*), as shown in the inset of Figure 1b. Again, it is important to note that the slightly higher *M_S_* values for CFA films grown on MgO over the whole *t_Pt_* range and the slower asymptotic saturation of *M_S_* for systems grown on Si. A phenomenological fit of *M_S_* versus *t_Pt_* with the equation $M_{S}=M_{S0}+A\left(1-e^{-\frac{t_{Pt}}{\lambda }}\right)$, shown in the inset of Figure 1b, allows for the deduction of the magnetization at saturation free of PIM (*M_S0_*) of 1000 emu/cm^3^ and the extent of PIM A = 100 emu/cm^3^. This corresponds to a change of 10% in the CFA magnetization for both systems, and a characteristic saturation thickness λ of 1 nm and 3.7 nm for films grown on MgO and Si, respectively.

### 3.2. Gyromagnetic Ratio

**Figure 2:**
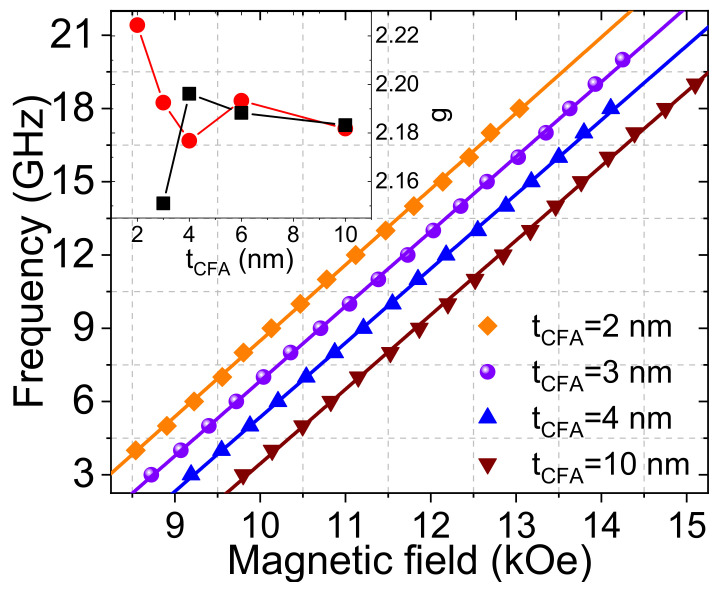
Variations in the uniform precession mode frequency versus the perpendicular to the film plane applied magnetic field for the Pt (4 nm)/Co_2_FeAl (*t_CFA_*)/MgO grown on Si substrate. Symbols refer to experimental data and solid lines are fits using Equation (1) of reference [18]. The inset shows the g-factor as a function of the *t_CFA_* for Pt (4 nm)/Co_2_FeAl (*t_CFA_*)/MgO grown on MgO (black curve) and Si (red curve) substrates.

In addition, *M_s,_* the g-factor (which is related to the gyromagnetic ratio (γ/2π = g × 13.97 GHz/T)) is a crucial parameter for enhancing the accuracy of the determination of the damping and the iDMI strength. It can be precisely determined from investigating the uniform precession mode frequency versus the perpendicular to the film plane applied magnetic field measured by MS-FMR technique, as shown in Figure 2 for films grown on Si substrate. According to Equation (1) of reference [18], γ/2π is straightforwardly determined from the slope of the linear fit of the experimental data presented in Figure 2. The obtained g-factor values are shown in the inset of Figure 2, where a slow thickness variation of g with similar values for both systems beyond 3 nm can be noticed. Furthermore, one should note the higher g values for thinner CFA films grown on Si due to the different surface and interface quality between the two systems.

### 3.3. Magnetic Damping

**Figure 3:**
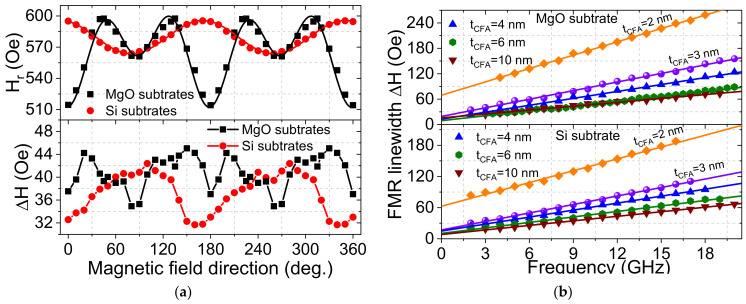
(**a**) Resonance field (*H_r_*) and half linewidth at half maximum (∆*H*) versus the direction of the in-plane applied magnetic field with a respect to the substrate edges measured at 7 GHz microwave frequency for Pt (4 nm)/Co_2_FeAl (6 nm)/Pt grown on Si or MgO substrate. Symbols refer to experimental data and solid lines (for *H_r_*) are fits using Equation (2) of reference [23]. (**b**) Variation of ∆*H* versus the microwave frequency for Pt (4 nm)/Co_2_FeAl (*t_CFA_*)/Pt grown on Si or MgO substrate. The in-plane magnetic field was applied along the direction giving the minimal ∆*H*. Symbols refer to experimental data and solid lines are fits using Equation (1).

The FMR linewidth gives information about the magnetization relaxation and samples inhomogeneities. Indeed, it results from intrinsic contributions which are isotropic (when magnetization is parallel to the applied magnetic field) and extrinsic mechanisms which are generally anisotropic. An efficient procedure to minimize the extrinsic contributions is to measure the in-plane angular dependence of ∆*H* to find out the direction of the in-plane applied magnetic field, giving the minimum value of the linewidth. The angular dependences of *H_r_* and ∆*H* were recorded at microwave driving frequency of 7 GHz where their typical behaviors are shown in Figure 3a for a 6 nm thick CFA layer grown on Si or MgO substrate. While the in-plane angular behavior of *H_r_* is governed by a uniaxial anisotropy not exceeding 20 Oe over the *t_CFA_* range for samples grown on Si, that of CFA grown on MgO is a superposition of uniaxial and dominant fourfold anisotropies which is thickness dependent. Due to the magnetocrystalline origin of the fourfold anisotropy as their principal directions agree with the epitaxial symmetry [20,23], we conclude on the full epitaxial growth of CFA on MgO as mentioned above. The in-plane angular dependence of ∆*H* shows a fourfold (four maxima) or uniaxial symmetry, reflecting the in-plane symmetry as it is observed for the angular dependence of *H_r_* for films grown on MgO or Si, respectively. Such behavior is a typical of anisotropic two-magnon scattering. Note the field dragging, which could induce such anisotropic behavior of ∆*H*, plays a minor role here. It is thus negligible in our samples since the in-plane anisotropy fields are significantly small with a respect to the resonance fields.

To precisely determine the magnetic damping constant, the frequency dependence of ∆*H* was investigated within the available microwave frequency range of 2–20 GHz (as shown in Figure 3b) for both systems. The in-plane magnetic field was applied along the direction that minimized ∆*H*. For all the samples, ∆*H* is linear with the microwave frequency, suggesting that it is due to the Gilbert mechanism. Therefore, ∆*H* can be fitted with Equation (1) to obtain α and the inhomogeneous linewidth (residual linewidth at zero microwave frequency: *f* = 0) ∆*H*_0,_ representing the frequency independent ∆*H*.

(1) $$\Delta H=\Delta H_{0}+\frac{2\pi }{\gamma }\alpha f,$$

The spin transport across interfaces in HM/FM systems, which is important in the development of future spintronic devices, can be characterized by the effective spin-mixing conductance ($g_{eff}^{↑↓}$). This parameter plays a crucial role during the spin pumping process and can be typically determined by measuring the FM thickness dependence of α. Based on a standard model, the FM thickness dependence attributed only to the enhancement of α by spin pumping into the HM layer is given by Equation (2).

(2) $$\alpha =\alpha_{CFA}+\frac{g\mu_{B}}{4\pi M_{S}t_{CFA}}g_{eff}^{↑↓},$$

where *µ_B_* is the Bohr magneton and *α_CFA_* is the Gilbert damping constant of the CFA.

**Figure 4:**
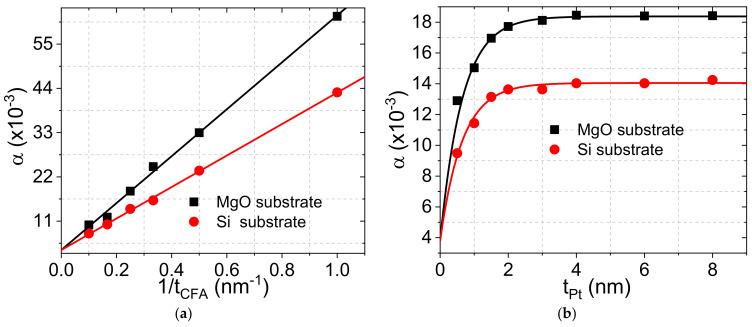
(**a**) Damping parameter (α) as a function of the CFA reciprocal thickness of Pt (4 nm)/Co_2_FeAl (*t_CFA_*)/MgO systems grown on Si or MgO substrate. Symbols refer to experimental data and solid lines are fits using Equation (2). (**b**) Variation of the damping constant versus the Pt thickness of Pt (*t_Pt_*)/Co_2_FeAl (4 nm)/MgO systems grown on Si or MgO substrate. Symbols refer to experimental data and solid lines are fits using Equation (3).

The variations of α versus the inverse of *t_CFA_*, shown in Figure 4a, reveal a linear dependence of α with 1/*t_CFA_* as expected from the spin pumping model. Using Equation (2), the Gilbert damping constant of the bulk CFA is found to be similar for both systems (*α_CFA_* = 3.8 × 10^−3^), whereas the estimated effective spin mixing conductance of CFA grown on MgO ($g_{eff}^{↑↓}=39.26\pm 2\;nm^{-2}$) is significantly higher than $g_{eff}^{↑↓}$ of CFA deposited on Si ($g_{eff}^{↑↓}=25\pm 1nm^{-2}$). Furthermore, $g_{eff}^{↑↓}$ of films grown on Si is comparable to the values of Pt/Py [24] ($g_{eff}^{↑↓}=$ 32 nm^−2^) and Pt/CoFe [25] ($g_{eff}^{↑↓}=$ 25 nm^−2^). Since the spin-dependent transport property depends on the surface morphology, atomic diffusion, crystal structure, and other parameters, the higher $g_{eff}^{↑↓}$ of CFA grown on MgO is most probably due to its epitaxial growth on MgO. This epitaxial growth could enhance the spin asymmetry around the Fermi level and, thus, the spin injection efficiency. This suggests that CFA grown on MgO could be a promising material candidate as a spin injection source via spin pumping. 

Another key parameter for spin-dependent transport through the FM/HM interface which is crucial for spintronic devices is the spin diffusion length (λ_SD_). λ_SD_ can be determined by investigating the HM thickness dependence of damping using Equation (3). The experimental data of the *t_Pt_* dependence of α, shown in Figure 4b, have been fitted with Equation (3). The obtained value of λ_SD_ = 1.3 nm for both systems is in agreement with the one obtained for Pt/Py (1.8 nm) [23]. Again, note the higher values of α over the whole Pt thickness due to higher spin pumping efficiency of the epitaxial CFA.

(3) $$\alpha =\alpha_{CFA}+\frac{g\mu_{B}}{4\pi M_{S}t_{CFA}}g_{eff}^{↑↓}\left[1-e^{-\frac{2t_{Pt}}{\lambda_{SD}}}\right],$$

### 3.4. iDMI

We also investigated iDMI in both systems through the analysis of ∆*F* between spin wave frequencies corresponding to Stokes and anti-Stokes lines. Due to the weak iDMI values for the thicker CFA samples (above 3 nm), and since BLS measurements are time-consuming compared to MS-FMR, we limited the determination of the iDMI effective constant (*D_eff_*) to ∆*F* measurements at maximal spin wave vector *k_sw_* = 20.45 µm^−1^ (corresponding to an angle of incidence of 60°) without a crossed analyzer. This allows for the probing of bulk phonon and magnon Stokes and anti-Stokes lines. The acoustic waves are used to check the zero frequency setting and to enable frequency correction (in case of incorrect adjustment of the zero frequency) for a precise measurement of the iDMI constant [26]. 

**Figure 5:**
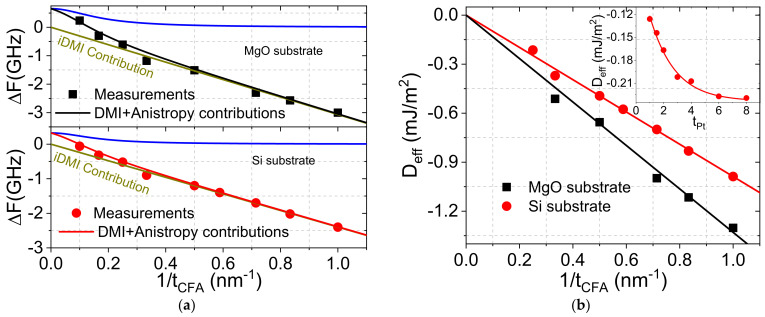
Variation of (**a**) the frequency shift between the Stokes and anti-Stokes lines (∆F) and (**b**) the effective iDMI constant as a function of the CFA reciprocal thickness of Pt (4 nm)/Co_2_FeAl (*t_CFA_*)/MgO systems grown on Si or MgO substrate. Symbols are experimental data while solid lines refer to fits using Equation (4) and Relation (8) of reference [28] for (**a**) and only Equation (4) for (**b**). The inset shows the variati ns of the effective iDMI constant (D*_eff_*) versus the Pt thickness for Pt/Co_2_FeAl (4 nm)/MgO) systems grown on Si. Symbols refer to experimental data while solid line is fit using $D_{eff}=D_{0}+D_{1}\left[1-e^{-\frac{t_{Pt}}{d}}\right]$.

Figure 5a shows the variations of ∆*F*, measured by BLS, versus 1/*t_CFA_* for both systems, where the observed linear behavior suggests that this non-reciprocity (∆*F*) is due to iDMI. However, a deviation from the linear behavior, which is more pronounced for films grown on MgO, can be observed for the thicker CFA films (above 3 nm), suggesting other contributions to ∆F. Furthermore, for the 10 nm thick CFA layer grown on MgO, ∆*F* is positive and cannot be due to iDMI since the latter should induce a negative frequency shift imposed by the negative sign of *D_eff_* of the Pt/CFA interface [27]. Indeed, asymmetry of the magnetic film properties due to an interface perpendicular magnetic anisotropy is known to induce spin wave non-reciprocity [28]. Therefore, iDMI always combines with the effect of the interface anisotropy difference because both contributions to the spin wave frequency obey the same symmetry [28]. To precisely determine the magnitude of iDMI, it is therefore essential to take into account the contribution due to the difference in the interface anisotropy. According to Gladii et al. [28], the frequency non-reciprocity due to the interface anisotropy difference scales linearly with the wave vector and quadratically with the FM thickness for thicknesses of 20 nm and below. Since iDMI scales linearly with the inverse of FM thickness, ∆*F* of the thickest CFA layers (*t_CFA_* > 3 nm) results mostly from the difference in anisotropy between the lower (Pt/CFA) and upper (CFA/MgO) interfaces while it is induced by iDMI for the thinner CFA films. To separate the two contributions, we first consider ∆*F* for *t_CFA_* < 3 nm. D*_eff_* is then determined from the relation:

(4) $$\Delta F=\frac{2\gamma D_{eff}}{\pi M_{S}}k_{sw}=\frac{2\gamma D_{s}}{\pi M_{S}t_{CFA}}k_{sw},$$

where *D_s_* is the iDMI surface constant, characterizing the iDMI strength and *k_sw_* = 20.45 µm^−1^. 

The obtained inverse CFA thickness dependence of *D_eff_*, shown in Figure 5b, allowed the *D*_s_ to be determined for both systems from the slope of the linear fit as indicated by Equation (4). Similar to $g_{eff}^{↑↓}$, *D_s_* of CFA films grown on MgO (*D_s_* = −1.33 pJ/m) is significantly higher than that of those deposited on Si, most probably due to the higher interface quality of the epitaxial CFA films. Once *D_s_* is determined, the data representing the variation of the total ∆*F* as a function of 1/*t_CFA_* were fitted using Equation (4) and the Relation (8) in reference [28], giving the analytical expression of the interface anisotropy difference induced non reciprocity. This allowed for the deduction of the difference in the interface anisotropy constants of the two interfaces with CFA, which was found to be: ${\left(K_{s}^{Pt/CFA}-K_{s}^{CFA/MgO}\right)}_{Si}$ = 0.33 erg/cm^2^ and ${\left(K_{s}^{Pt/CFA}-K_{s}^{CFA/MgO}\right)}_{MgO}$ = 0.7 erg/cm^2^.

To separate the contribution of each interface with CFA (Pt/CFA and CFA/MgO) to the perpendicular magnetic anisotropy, we combined the above-mentioned values of the interface anisotropy constants’ difference between the top and bottom interfaces with the effective anisotropy constant ($K_{s}=K_{s}^{Pt/CFA}+K_{s}^{CFA/MgO}$) values. The latter is obtained for each system through the measurement of the effective magnetization deduced from the FMR measurements of the thicker film (above 3 nm) as a function of 1/*t_CFA_* (not shown here). The obtained values are found to be K_s_ = 0.57 erg/cm^3^ and K_s_ = 0.41 erg/cm^3^ for CFA grown on MgO or Si, respectively. As a result, the deduced values of $K_{s}^{CFA/MgO}$ are found to be negligible for both systems ($K_{s}^{CFA/MgO}=0.04$ erg/cm^2^ and −0.06 erg/cm^2^ for systems grown on Si or MgO, respectively). In contrast, $K_{s}^{Pt/CFA}$ of CFA deposited on MgO (0.63 erg/cm^2^) is significantly higher than that of samples on Si (0.37 erg/cm^2^), confirming again the higher quality of the CFA grown on MgO due to epitaxial growth. We should mention that since the interface anisotropy difference can only be evidenced for the thicker CFA films (*t_CFA_* > 3 nm), the separation of the individual contributions of each interface to the perpendicular anisotropy is only valid for this range of thicker CFA films.

The inset of Figure 5b shows the *t_Pt_* dependence of *D_eff_* for CFA-based systems grown on Si. *D_eff_* increases drastically (in absolute value) with *t_Pt_* for thinner Pt layer (below 3 nm) before a saturation for thicker Pt. To determine the characteristic saturation thickness (*d*) of *D_eff_*, the experimental data were fitted with the phenomenological relation $D_{eff}=D_{0}+D_{1}\left[1-e^{-\frac{t_{Pt}}{d}}\right]$, where *D*_0_, *D*_1_, and *d* are the fitting parameters. *D*_0_ refers to D*_eff_* in absence of Pt and *D*_1_ measures the extent of iDMI. *D*_0_ is expected to be very weak since Pd and MgO are expected to induce negligible iDMI [26]. The obtained values are *d* = 1.82 nm, *D*_0_ = −0.04 erg/cm^2^ and *D*_1_ = −0.2 erg/cm^2^ and, therefore, the iDMI saturation value for 4 nm thick CFA layer is *D*_1_ + *D*_0_ = −0.24 erg/cm^2^. This gives *Ds* = −0.96 pJ/m, which is in agreement with the obtained value from the CFA thickness dependence of *D_eff_*. The characteristic saturation thickness is in agreement with those obtained for Pt/Co-based systems (2.4 nm) [29] and is also comparable to the above-obtained value of λ_SD_. Since the iDMI strength is related to the spin–orbit coupling parameter, it is reasonable that only Pt atoms placed within the spin diffusion length can interact with CFA atoms [29]. The *t_Pt_* behavior of *D_eff_* could be due to cumulative electron hopping at the interface between Pt atoms and CFA atoms or to structural changes as Pt thickness varies [29].

## 4. Conclusions

Co_2_FeAl (CFA) thin films of variable thickness (1 nm ≤ *t_CFA_* ≤ 10 nm) were grown on a Si or MgO substrate using a MgO capping layer and a Pt buffer layer of various thicknesses (0.5 nm ≤ *t_Pt_* ≤ 8 nm). A vibrating sample magnetometer was used to investigate the thickness dependence of the magnetic moment at saturation and revealed a zero-magnetic dead layer and higher magnetization at saturation of CFA films grown on MgO. Microstrip ferromagnetic resonance has been used to investigate the spin-pumping-induced damping. The in-plane angular dependences of the resonance field and the filed linewidth revealed extrinsic magnetization relaxation contributions. It also allowed for the conclusion that the in-plane magnetic anisotropy is governed by a uniaxial or a fourfold term superimposed to a uniaxial anisotropy for samples grown on Si or MgO, respectively. This implies a polycrystalline or an epitaxial growth for CFA deposited on Si or MgO, respectively. Due to their epitaxial character, CFA on MgO exhibited strong iDMI and higher spin mixing conductance, making them promising candidates for spin injection sources via spin pumping and for other spintronic applications.

## Data Availability

Data are available upon request.
